# Increased peritoneal B1-like cells during acute phase of human septic peritonitis

**DOI:** 10.1016/j.isci.2024.110133

**Published:** 2024-06-06

**Authors:** Christian von Loeffelholz, René Winkler, Cynthia Weigel, Eva-Maria Piskor, Wolfgang Vivas, Falk Rauchfuß, Utz Settmacher, Ignacio Rubio, Sebastian Weis, Markus H. Gräler, Michael Bauer, Christian Kosan

**Affiliations:** 1 Department of Anesthesiology and Intensive Care, Jena University Hospital, Friedrich Schiller University, Am Klinikum 1, 07749 Jena, Germany; 2 Department of Biochemistry, Center for Molecular Biomedicine (CMB), Friedrich Schiller University, Hans-Knöll-Str. 2, 07745 Jena, Germany; 3 Center for Molecular Biomedicine (CMB), Friedrich Schiller University, Hans-Knöll-Str. 2, 07745 Jena, Germany; 4 Leibniz Institute for Natural Product Research and Infection Biology, Hans Knöll Institute (HKI), 07745 Jena, Germany; 5 Department of General, Visceral and Vascular Surgery, Jena University Hospital, Am Klinikum 1, 07749 Jena, Germany; 6 Institute of Infectious Disease and Infection Control, Friedrich Schiller University, Am Klinikum 1, 07749 Jena, Germany; 7 Center for Sepsis Control and Care (CSCC), Jena University Hospital, Am Klinikum 1, 07749 Jena, Germany

**Keywords:** Biological sciences, Immune response, Immunology

## Abstract

Sepsis is a life-threatening condition caused by dysregulated host responses to infection. Myeloid cell accumulation and lymphocyte decline are widely recognized phenomena in septic patients. However, the fate of specific immune cells remains unclear. Here, we report the results of a human explorative study of patients with septic peritonitis and patients undergoing abdominal surgery without sepsis. We analyzed pairwise peritoneal fluid and peripheral blood taken 24 h after surgery to characterize immediate immune cell changes. Our results show that myeloid cell expansion and lymphocyte loss occur in all patients undergoing open abdominal surgery, indicating that these changes are not specific to sepsis. However, B1-like lymphocytes were specifically increased in the peritoneal fluid of septic patients, correlating positively with sequential organ failure assessment (SOFA) and acute physiology and chronic health evaluation II (APACHE-II) clinical severity scores. In support of this notion, we identified an accumulation of peritoneal B1b lymphocytes in septic mice.

## Highlights

• Abdominal surgery induces severe immune cell changes independent of sepsis
• Increased CD5^+^ B1-like cell numbers in peritoneal fluids are indicative of sepsis
• Clinical severity correlates with B1-like cell frequencies in humans and mice
• Murine B1a cells are depleted and B1b cells are maintained during sepsis

## Graphical abstract

**Figure:**
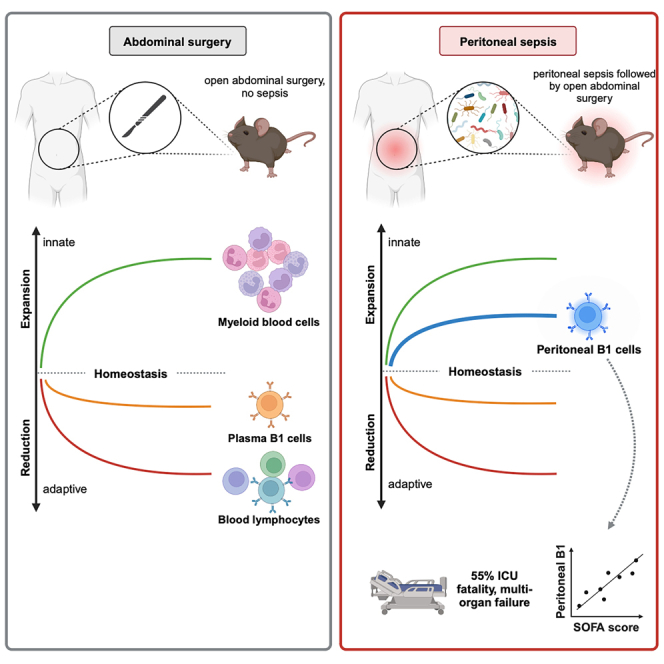

## Introduction

Sepsis is a condition that is recognized as a leading cause of critical illness and mortality worldwide.^1^^,^^2^^,^^3^ Sepsis results from an inappropriate host response to an infecting pathogen. Resulting cell and organ dysfunction (OD) or multiple organ failure (MOF) define complications of sepsis and are categorized by the sequential organ failure assessment (SOFA) score.^2^^,^^4^ The acute physiology and chronic health evaluation II (APACHE-II) score is routinely used in the intensive care setting to estimate outcomes.^5^ Early phases of sepsis pathophysiology can be characterized by hyper-inflammatory processes, driven mainly by innate immune cell populations, representing the first line of defense.^6^^,^^7^ Simultaneously, acute immune suppression is frequently observed in sepsis, primarily affecting adaptive immunity and contributing to prolonged critical illness and delayed mortality.^8^^,^^9^^,^^10^^,^^11^^,^^12^^,^^13^

Early circulatory shock, OD, and MOF can also result from a sterile systemic inflammatory reaction due to trauma, burns, or surgery, a setting formerly referred to as systemic inflammatory response syndrome.^2^^,^^8^^,^^9^^,^^10^^,^^11^^,^^14^^,^^15^ However, this sterile inflammatory reaction can reflect an appropriate host response and, therefore, needs to be discriminated from sepsis.^2^ Data from trauma or surgery patients suggest that mainly T cell numbers are reduced under such conditions, and prolonged lymphopenia for more than three days is correlated with higher morbidity and mortality.^12^^,^^13^^,^^16^^,^^17^^,^^18^^,^^19^^,^^20^

Current research on sepsis-associated lymphopenia has focused on T cells, while the fate and role of B lymphocytes are much less understood. Among all types of B cells, B1-like cells represent a subclass with innate-like properties that significantly set them apart from the conventional B2 lymphocyte.^21^ The relatively small but unique subset of B1-like cells derives from progenitor cells in the fetal liver during embryonic development.^22^ B1 cells were first described in mice and represent only 1%–2% of all B cell populations in the spleen but 30%–70% in the pleura and peritoneum to provide immediate defense against microbial agents.^21^^,^^23^ In mice, B1 cells are classified based on the surface expression of CD5 into B1a (CD5^+^) and B1b (CD5^−^). B1a cells are often considered to be phenotypically more similar to conventional B2 B lymphocytes, while from a functional viewpoint, some authors suggest that only B1b cells generate a T cell-independent memory response.^21^^,^^24^ In humans, all CD5-expressing B cells are considered to be B1-like.^25^ B1-like cells express low-affinity natural antibodies with broad specificity, which are capable of recognizing molecules expressed by microbial agents and play a critical role in rapid immune responses to infections in neonatals.^21^ Importantly, B1-like B cells have not been studied in adult peritoneal sepsis.

In this study, we enrolled patients with secondary peritonitis due to sepsis who underwent open abdominal emergency surgery for therapeutic indication. This allowed us to keep the group of septic patients studied as homogeneous as possible and analyze peritoneal fluids in the early postoperative phase. To control for the effect of surgical intervention as an inflammation-inducing insult with potential effects on host immunity,^26^ we additionally included age- and gender-matched patients undergoing elective open abdominal surgery for non-infectious/non-inflammatory indication. Moreover, we investigated three distinct mouse models of sepsis regarding their ability to reflect human septic peritonitis. For our study, we obtained data from human and murine samples to investigate the fate of B lymphocytes, including B1-like cells, during sepsis.

## Results

### A matching human patient cohort to study sepsis-related changes

To obtain data on acute human sepsis, we conducted an explorative trial on three distinct cohorts: patients with sepsis requiring abdominal surgery, patients without sepsis undergoing abdominal surgery, and healthy control individuals. Sepsis was defined according to recognized clinical criteria: a life-threatening OD caused by a dysregulated host response, identified by an acute change in SOFA score ≥2 points consequent to the infection.^2^ The leading cause of surgery in all septic patients was peritonitis due to an underlying surgically treatable source of infection. Therefore, all our included sepsis patients homogenously suffered from septic peritonitis. Patients and healthy controls were comparable in age and gender distribution (Table 1). Microbiological analysis indicated an infectious agent for all patients with sepsis requiring surgery, while no cultured microbial infection was traceable in patients undergoing elective surgery. Baseline laboratory indices of systemic inflammation like C-reactive protein or white blood cell count were not significantly different between the surgery-only and sepsis group during the acute phase of inflammation. Septic patients had a higher rate of postoperative mechanical ventilation, renal replacement therapy, vasopressor therapy, and transfusion and significantly elevated bilirubin, creatinine, and lactate which is in accordance with the presence of OD. The criteria for septic shock,^2^ i.e., persisting hypotension requiring vasopressors to maintain mean arterial blood pressure ≥65 mmHg and serum lactate levels >2 mmol/L despite adequate volume resuscitation were met by 78% of peritonitis patients. Consequently, sepsis patients had markedly higher SOFA and APACHE-II scores than surgery-only controls, a longer intensive care unit (ICU) stay, and an ICU mortality of 55% compared to no fatalities in the surgery-only group. Besides, rates of type 2 diabetes, a major co-morbidity and risk factor for endothelial and immune dysfunction,^27^^,^^28^ were comparable between sepsis and surgery-only patients.

**Table 1:** Clinical characteristics of the study group

Parameter	Sepsis	Surgery	Control	*p* value
n (male)	9 (6)	12 (9)	7 (6)	0.68
Age (years)	69 ± 6	61 ± 5	60 ± 5	0.23
Baseline APACHE-II score	24.6 ± 2.8	9.9 ± 3.3	–	0.010
Baseline SOFA score	11.9 ± 0.5	3.8 ± 1.1	–	<0.001
Evidenced microbial culture (n)	9	0	–	<0.001
Septic shock (n)	7	0	–	<0.001
ICU stay after surgery (n)	9	7	–	<0.001
ICU stay (d)	27.2 ± 3.7	0.9 ± 0.4	–	<0.001
Vasopressor (n)	9	4	–	0.002
Invasive ventilation (n)	9	5	–	0.005
Renal replacement therapy (n)	2	0	–	0.11
Transfusion (n)	5	0	–	0.003
ICU fatality (n)	5	0	–	0.003
Baseline CRP (mg/L)	188.3 ± 38.3	101.5 ± 25.5	–	0.21
Baseline WBC (x10^3^/μL)	18.3 ± 7.8	15.2 ± 1.1	–	0.18
Baseline lactate (mmol/L)	5.5 ± 1.4	2.0 ± 0.4	–	0.002
Baseline creatinine (μmol/L)	166.3 ± 31.3	86.8 ± 8.8	–	0.022
Baseline bilirubin (μmol/L)	27.2 ± 5.7	13.4 ± 2.3	–	0.013
Baseline glucose (mmol/L)	8.3 ± 0.5	9.0 ± 0.6	–	0.55
Baseline albumin (g/L)	17.4 ± 1.4	25.7 ± 4.5	–	0.073
Malignancy (n)	2	9	0	0.002
Type 2 diabetes (n)	4	4	0	0.13

Data are given as mean ± SEM or absolute numbers. Differences between groups were evaluated by using the Kruskal-Wallis or Mann-Whitney procedure, nominal variables by the chi-squared test.

APACHE, acute physiology, and chronic health evaluation; CRP, C-reactive protein; ICU, intensive care unit; n, numbers; SOFA, sequential organ failure assessment; WBC, white blood cell count.

### Lymphopenia and myelocytosis are characteristics of severely ill patients suffering from sepsis or surgery-induced inflammation

**Figure 1:**
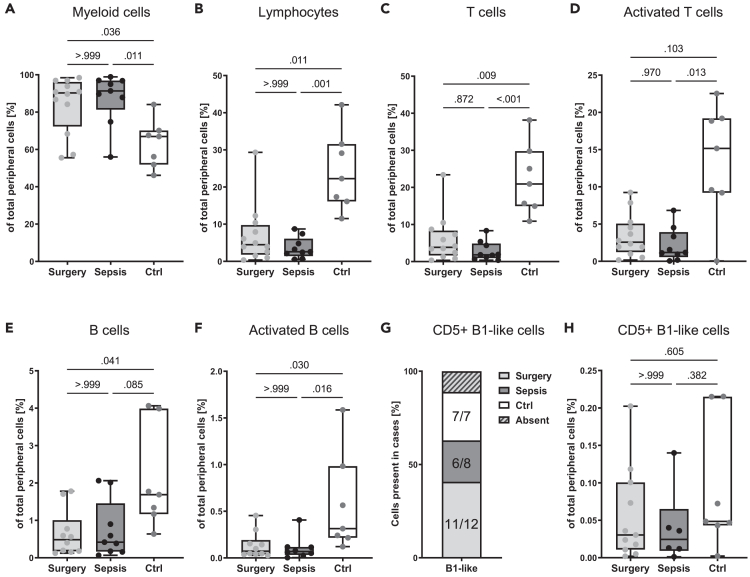
Lymphopenia and myelocytosis are characteristics of severely ill patients suffering from sepsis or surgery-induced inflammation (see also Figures S1 and S2) Peripheral venous blood samples from septic patients, patients with surgery-induced sterile inflammation during the acute phase of major systemic inflammation, and healthy volunteers (Ctrl) was subjected to flow cytometric immune phenotyping. (A) Myeloid cell (CD11b^+^), (B) total lymphocyte (CD19^+^ and CD3^+^ cells), (C) T cell (CD19^−^CD3^+^), (D) activated T cell (CD19^−^CD3^+^CD27^+^), (E) B cell (CD3^−^CD19^+^), and (F) activated B cell (CD3^−^CD19^+^CD27^+^) populations were analyzed. (G) The presence of B1-like cells (CD3^−^CD19^+^CD5^+^) in peripheral blood samples is shown. Absence corresponded to zero events measured in the indicated gates. (H) The percentage of B1-like cells (CD3^−^CD19^+^CD5^+^) in the peripheral blood was measured. *n* = 7–12 per group. Each dot represents one individual patient. (A)–(F), (H) Boxes in all boxplots span from 25^th^-75^th^ percentile and whiskers indicate the minimum and maximum values inside the 1.5-times interquartile range above and below first and third quartiles. Kruskal-Wallis test with *post hoc* Dunn’s correction.

We collected peripheral venous blood samples for flow cytometry from patients during the acute phase of systemic inflammation (24 h post-surgery) as well as from healthy volunteers (Ctrl). Compared to healthy controls, both sepsis and surgery-only patients had increased myeloid cell counts in the blood under conditions of acute systemic inflammation (Figure 1A), while T cells and B cells decreased (Figures 1B–1F). However, we found no significant differences in myeloid (CD11b^+^), total lymphocyte (CD19^+^ and CD3^+^ cells), T cell (CD19^−^CD3^+^), activated T cell (CD19^−^CD3^+^CD27^+^), B cell (CD3^−^CD19^+^), and activated B cell (CD3^−^CD19^+^CD27^+^) populations between sepsis and surgery-only patients. Only activated T cells (CD19^−^CD3^+^CD27^+^) were significantly reduced in the peripheral blood of sepsis patients compared to healthy controls but not in surgery-only patients (Figure 1D). We also analyzed the presence of circulating B1-like B cells (CD3^−^CD19^+^CD5^+^) using a specific gating strategy (Figure S1). B1-like cells were found in the peripheral blood of all healthy controls and more than 75% of patients (Figure 1G). However, we detected no significant differences in the percentage of B1-like cells among our cohorts (Figure 1H). Therefore, we conclude that CD5^+^ B1-like cells appear to be more resistant to the acute phase of systemic inflammation than other adaptive immune cell populations, as seen by their constant presence in peripheral blood. These results highlight the complex changes in the immune cell population during systemic inflammation and suggest that B1-like cells may play a unique role in this process.

### B1-like lymphocytes are increased in peritoneal fluids from septic patients

**Figure 2:**
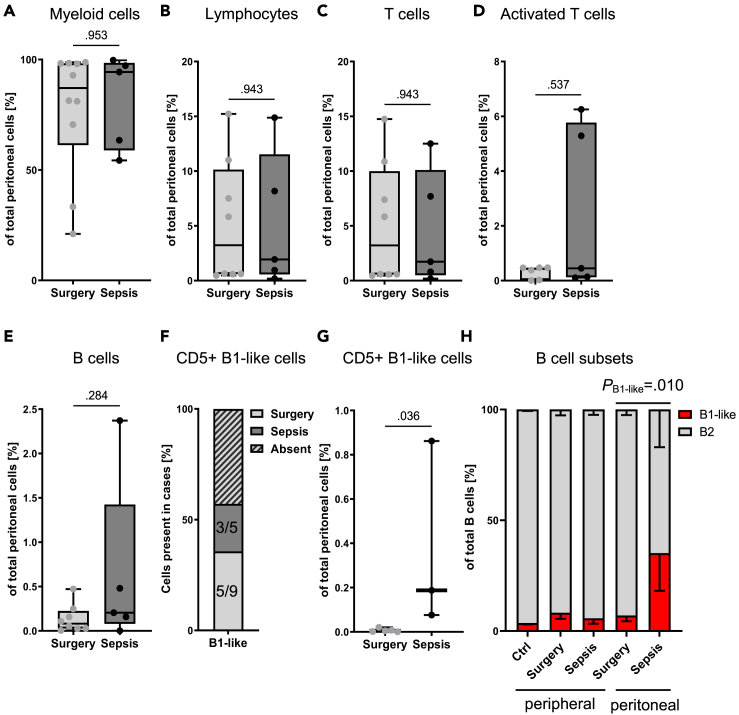
B1-like lymphocytes are increased in peritoneal fluids from septic patients Peritoneal fluids sampled within 24 h after open abdominal surgery from patients suffering from sepsis due to secondary peritonitis or surgery-only patients were subjected to flow cytometric immune-phenotyping. (A) Myeloid cell (CD11b^+^), (B) total lymphocyte (CD19^+^ and CD3^+^ cells), (C) T cell (CD19^−^CD3^+^), (D) activated T cell (CD19^−^CD3^+^CD27^+^), and (E) B cell (CD3^−^CD19^+^) populations were analyzed. (F) The presence of B1-like cells (CD3^−^CD19^+^CD5^+^) in peritoneal fluid samples is shown. Absence corresponded to zero events measured in the indicated gates. (G) The percentage of B1-like cells (CD3^−^CD19^+^CD5^+^) in the peritoneal fluid was measured. (H) The percentage of B1-like and B2 cell populations among total B cells is shown under the indicated conditions. Bars depict mean ± SEM. Two-way ANOVA with *post hoc* Tukey’s correction. *n* = 5–10 per group. Each dot represents one individual patient. (A)–(E), (G) Boxes in all boxplots span from 25^th^-75^th^ percentile and whiskers indicate the minimum and maximum values inside the 1.5-times interquartile range above and below first and third quartiles. Mann-Whitney U test.

To understand the impact of severe peritonitis on local immune cell populations in the peritoneal cavity, we analyzed human peritoneal lavage samples taken up to 24 h after open abdominal surgery from the same patients. Leukocytes were enriched using positive magnetic selection with CD45 to reduce bacterial contamination and facilitate the analysis of adaptive immune cells. Using flow cytometry, we examined myeloid cells, total lymphocytes, T cells, activated T cells, and B cells in peritoneal fluids as defined earlier (Figures 2A–2E). We did not detect significant differences between sepsis and non-septic surgery patients in these immune cell populations. CD5^+^ B1-like lymphocytes were present in the majority of sepsis and surgery-only cases (Figure 2F). The most intriguing discovery, however, was the robust increase of B1-like lymphocyte percentages in peritoneal fluids of sepsis patients (*p* = 0.036; Figure 2G). The increased frequency of B1-like cells among B cells was unique to the peritoneal fluids of septic patients and not found in the surgery-only group or peripheral blood of septic patients (Figure 2H). These findings imply that during the early phase of sepsis-induced peritonitis, B1-like lymphocytes are either recruited to the infection site or do not succumb to sepsis-associated lymphopenia.

### B1-like cell frequencies in the peritoneal fluid correlate with the clinical course in humans

**Figure 3:**
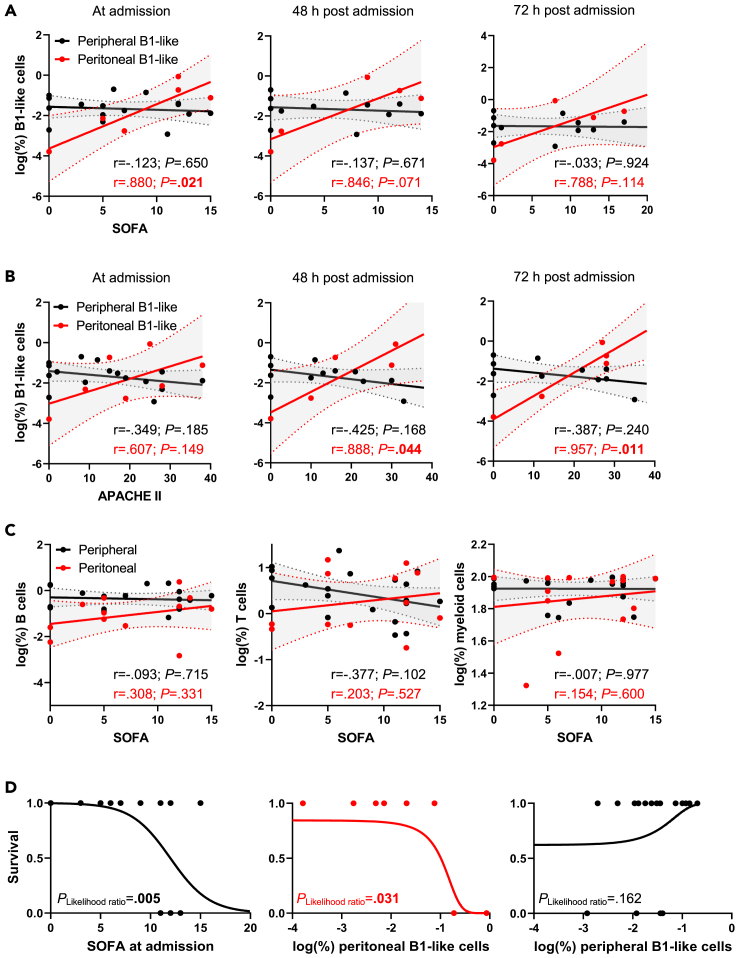
B1-like cell frequencies in the peritoneal fluid correlate with the clinical course in humans Percentages of peritoneal and peripheral B1-like cells were logarithmically transformed and correlated with (A) the SOFA or (B) the APACHE-II score for the indicated time points of each patient. Pearson's correlation coefficient (r) was used for correlation analysis, and 95% confidence intervals are shaded. (C) SOFA score at admission time point was correlated with logarithmically transformed B cell, T cell, or myeloid cell percentages. Pearson's correlation coefficient (r) was used for correlation analysis, and 95% confidence intervals are shaded. (D) Simple logistic regression of SOFA at admission, peritoneal B1-like cell percentages, and peripheral B1-like cell percentages with survival outcome was performed. The *p* value from the likelihood ratio test is indicated.

Given the observed variations in peritoneal CD5^+^ B1-like cell numbers, we wondered if peritoneal B1-like cells qualified as predictors of prognosis for ICU patients. We correlated B1-like cells with the SOFA score at ICU admission (Figure 3A) and obtained a strong and significant correlation for peritoneal B1-like cells (r = 0.880; *p* = 0.021) but not B1-like cells from peripheral blood (r = −0.123; *p* = 0.650). The correlation between SOFA and peritoneal B1-like cells was only present during the early postoperative stage in sepsis but not at later time points (Figure 3A). Moreover, peritoneal B1-like lymphocyte frequencies also positively correlated with the APACHE-II score (r = 0.888; *p* = 0.044 and r = 0.957; *p* = 0.011 at 48 and 72 h, respectively) after ICU admission (Figure 3B). Interestingly, no significant correlation was found between the SOFA score and the numbers of CD19^+^ B cells, CD3^+^ T cells, or CD11b^+^ myeloid cells in peripheral blood or peritoneal fluid (Figure 3C). Our analysis also revealed that B cell or T cell proportions in the blood had no predictive value regarding ICU patient survival (Figures S2A and S2B). Simple logistic regression showed that peritoneal B1-like cells predicted survival in human patients (*p* = 0.031), like the established SOFA score (*p* = 0.005; Figure 3D). Taken together, these results suggest that the abundance of B1-like cells in the peritoneal fluid may reflect clinical severity and disease course in patients with sepsis or abdominal surgery.

### A comparison of experimental sepsis models to study peritoneal B cell-specific changes

**Figure 4:**
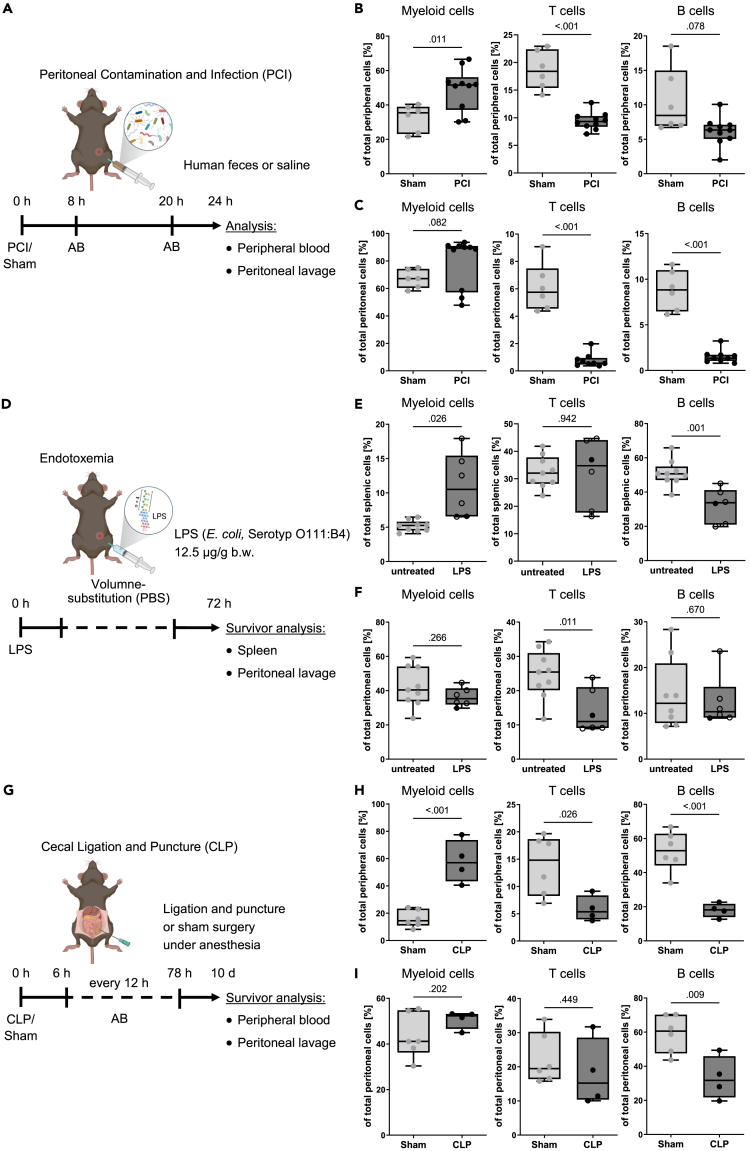
Immune cell composition in murine sepsis (see also Figures S3–S5) (A) A peritoneal contamination and infection model (PCI). Sepsis was induced in mice by i.p. injection of a human stool suspension. Sham animals received vehicle. Treatment with the antibiotic (AB) meropenem was started 8 h after infection and repeated every 12 h. Immune cells were analyzed after 24 h. Created with BioRender.com. (B) The percentage of CD11b^+^ myeloid cells, CD3^+^ T cells, and CD19^+^ B cells was analyzed in the peripheral blood by flow cytometry. *n* = 5–10 per group. Each dot indicates one animal. Unpaired, two-tailed t test with Welch’s correction. (C) The percentage of CD11b^+^ myeloid cells, CD3^+^ T cells, and CD19^+^ B cells was analyzed in the peritoneal lavage fluid. *n* = 5–10 per group. Each dot indicates one animal. Unpaired, two-tailed t test with Welch’s correction where required. (D) An endotoxemia model. Endotoxemia was induced in mice by intraperitoneal injection of 12.5 μg/g body weight purified lipopolysaccharide (*E. coli* serotype O111:B4). Survivors were analyzed after 3 days. Created with BioRender.com. (E) The percentage of CD11b^+^ myeloid cells, CD3^+^ T cells, and CD19^+^ B cells was analyzed in the spleen by flow cytometry. *n* = 6–9 per group. Each dot indicates one animal. Open dots indicate mice that were deficient for *Aicda*. Unpaired, two-tailed t test with Welch’s correction where required. (F) The percentage of CD11b^+^ myeloid cells, CD3^+^ T cells, and CD19^+^ B cells was analyzed in the peritoneal lavage fluid. *n* = 6–9 per group. Each dot indicates one animal. Open dots indicate mice that were deficient for *Aicda.* Unpaired, two-tailed t test. (G) A cecal ligation and puncture model (CLP). Sepsis was induced in mice by the outlined procedure. Sham animals received anesthesia and sham surgery without ligation/puncture. Treatment with the antibiotics (AB) imipenem/cilastatin was started 6 h after infection and repeated twice daily for three days. Survivors were analyzed after 10 days. Created with BioRender.com. (H) The percentage of CD11b^+^ myeloid cells, T cells (sum of CD4^+^ and CD8^+^), and CD19^+^ B cells was analyzed in the peripheral blood by flow cytometry. *n* = 4–6 per group. Each dot indicates one animal. Unpaired, two-tailed t test. (I) The percentage of CD11b^+^ myeloid cells, T cells (sum of CD4^+^ and CD8^+^), and CD19^+^ B cells was analyzed in the peritoneal lavage fluid by flow cytometry. *n* = 4–6 per group. Each dot indicates one animal. Unpaired, two-tailed t test. Boxes in all boxplots span from 25^th^-75^th^ percentile and whiskers indicate the minimum and maximum values inside the 1.5-times interquartile range above and below first and third quartiles.

Lymphopenia is a common feature of sepsis in both humans and rodents. Therefore, we compared three different experimental murine models of sepsis to evaluate which model reflects human septic peritonitis best. First, we induced acute septic peritonitis in mice by intraperitoneal application of a diluted human feces suspension (peritoneal contamination and infection, PCI, Figure 4A). Blood and peritoneal fluid were collected 24 h post-induction. During this early phase of sepsis, innate immune cells from the myeloid compartment were found to be increased in the blood, while T cell populations were significantly reduced (Figure 4B). Although T cells and B cells were significantly decreased in the peritoneal cavity of mice receiving PCI compared to sham (Figure 4C), the absolute numbers of T cells and B cells remained unchanged (Figures S3A and S3B). Instead, a dramatic influx of myeloid cells, mostly macrophages, was observed in mice receiving PCI (Figures S3B and S3C). We conclude that the PCI model shows a selective decline of adaptive immune cells and concomitant rise of myeloid cells in the blood, similar to septic patients, but the infiltration of myeloid cells in the peritoneal cavity due to the microbial burden makes it difficult to analyze B cell-related changes.

Second, we investigated an endotoxemia mouse model in which purified lipopolysaccharide (LPS) from *E. coli* was injected intraperitoneally (Figure 4D). This model was characterized by high mortality, weight loss, and hypothermia (Figure S4A). We analyzed wild-type mice and activation-induced cytidine deaminase (AID)-knockout (KO) mice that were deficient for AID (*Aicda*^−/−^).^29^ AID-KO mice have functional T cells and their B cells do not produce high-affinity antibodies but secrete cytokines after activation.^30^ However, we did not detect any differences between wild-type and AID-KO mice in our endotoxemia model regarding survival or severity (Figure S4A) and decided to analyze survivors from both cohorts together due to the high mortality 72 h post-injection. Interestingly, LPS injection increased the frequency of myeloid cells and depleted B cells in the spleen but did not affect splenic T cells (Figure 4E). In contrast, peritoneal T cells were reduced after LPS injection, but peritoneal B cells and myeloid cells in the peritoneal cavity were unaffected (Figure 4F). We also analyzed cytokine levels in plasma and detected a strong release of tumor necrosis factor, interleukin-6 (IL-6), monocyte chemoattractant protein-1, and IL-10 48 h after endotoxemia (Figures S4B and S4C). At 72 h after endotoxemia, circulating cytokine amounts returned to basal levels (Figures S4B and S4C). Taken together, LPS induces a strong pro-inflammatory reaction and depletes splenic B cells, but it does not affect peritoneal B cells.

Third, we chose cecal ligation and puncture (CLP) as an alternative experimental sepsis mouse model (Figure 4G). Peripheral blood and peritoneal lavage fluid were analyzed in survivors on day 10. CLP caused severe peritonitis with low mortality, weight loss, slight hypothermia, and reduced peripheral glucose levels (Figure S5A). Importantly, CLP caused only a mild cell infiltration into the abdomen (Figures S5B and S5C). Similar to the other murine sepsis models, we observed a heavy expansion of myeloid cells in the peripheral blood, while adaptive immune cells dramatically dropped (Figure 4H). We further observed that absolute and relative numbers of T cells and myeloid cells remained similar to sham treatment in the peritoneal cavity (Figure 4I). Nevertheless, a change in myeloid subpopulations was noted (Figure S5D). Importantly, relative peritoneal B cell numbers were significantly decreased in the CLP cohort (Figure 4I). Taken together, these results underline that the CLP model is suitable for analyzing changes in the peritoneal B cell compartment without altering other peritoneal immune cell compartments.

### B1b cell subpopulations are prominent in the peritoneal cavity of murine sepsis survivors

**Figure 5:**
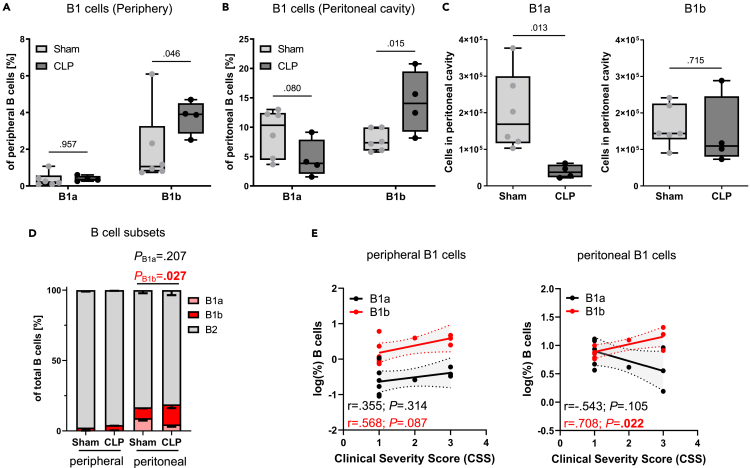
B1b cell subpopulations are prominent in the peritoneal cavity of murine sepsis survivors (see also Figures S6 and S7) B1 cells (CD19^+^B220^-^^/low^CD43^+^) in the CLP model (Figures 4F–4H) were further distinguished by CD5 into B1a (CD5^+^) and B1b (CD5^−^) for peripheral blood (A) and (B) peritoneal lavage fluid. *n* = 4–6 per group. Each dot indicates one animal. Two-way ANOVA. (C) Absolute cell numbers for B1a and B1b cells are shown. *n* = 4–6 per group. Each dot indicates one animal. Unpaired, two-tailed t test with Welch’s correction where required. (D) The percentage of B1 and B2 cell populations among total B cells is shown under the indicated conditions. Bars depict mean - SEM. Two-way ANOVA with *post hoc* Tukey’s correction. (E) Percentages of peripheral and peritoneal B1 cells were logarithmically transformed and correlated with the clinical severity score (CSS) 48 h post-CLP or sham. Pearson's correlation coefficient (r) was used for correlation analysis, and 95% confidence intervals are shaded. *n* = 4–6 per group. Each dot indicates one animal. (A)–(C) Boxes in all boxplots span from 25^th^-75^th^ percentile and whiskers indicate the minimum and maximum values inside the 1.5-times interquartile range above and below first and third quartiles.

We then continued to study B1 cells in murine sepsis by using the CLP model and applied a refined gating strategy to investigate B1 lymphocytes (Figure S6). Of note, B1 cells in mice are well defined (CD19^+^B220^−^^/low^CD43^+^) and can be subdivided into B1a (CD5^+^) and B1b (CD5^−^), which is different from humans.^23^^,^^25^ Importantly, we were not able to detect differences in the B1 subpopulations B1a and B1b in the peripheral blood of mice surviving from CLP or sham (Figure 5A). However, CD5-negative B1b cells were highly increased in the peritoneal cavity of septic mice (Figure 5B). The relative increase in B1b cells in the peritoneal cavity was potentially due to a significant drop in the total cell numbers of B1a cells (Figure 5C). This suggests that murine B1a cells disappear during sepsis, alike conventional B2 cells, and B1b cells remain present or reinfiltrate (Figure 5D). The depletion of B1a cells was also observed in the peritoneal cavity of mice receiving LPS injection, indicating a joint mechanism (Figures S7A–S7C).

Strikingly, the B1b cell numbers in the murine peritoneal cavity correlated positively with disease severity (r = 0.708; *p* = 0.022), as assessed with an established clinical severity score (Figure 5E). No significant correlation was obtained for peripheral B1 cells or peritoneal B1a cells (Figure 5E). These results show that experimental sepsis has a different outcome on the two subsets of B1 lymphocytes in mice.

## Discussion

With this explorative study, we characterized the acute phase of peritoneal sepsis and related open abdominal surgery on main immune cell populations in peritoneal fluids and peripheral blood of patients with proven sepsis and studied potential effects on CD5^+^ B1-like lymphocytes.

Approximately 30% of patients undergoing highly invasive surgical procedures develop sepsis, a complication associated in particular with post- or peri-operative lymphopenia.^31^^,^^32^ It is now increasingly recognized that not only insufficient nutrition protocols but also immunosuppression as reflected by persistent lymphopenia may play a critical role in the development of persistent inflammation, immunosuppression, and catabolism syndrome, which is related to long-term prognosis.^12^^,^^33^^,^^34^ In line with this concept, evidence from pre-clinical animal studies indicates that prevention of lymphopenia, for example by IL-7 administration, improves the long-term outcome of sepsis.^35^^,^^36^ All these observations highlight the importance of a better understanding of the pathophysiology of sepsis-associated lymphopenia in hospitalized critically ill subjects.^34^^,^^35^

Here, we provide data reporting the changes in immune cell populations in the peritoneal cavity during peritonitis-associated sepsis in mice and humans. Importantly, our comparison of septic patients with surgery versus surgery-only patients illustrates that the peritoneum-based response largely mirrors systemic changes (lymphopenia and myelocytosis) with one notable exception: we observed a relative increase in the B1-like cell population in peritoneal fluids of patients in the acute phase of septic peritonitis compared to patients with open abdominal surgery in the absence of bacterial infection. Furthermore, we observed a significant relationship of peritoneal B1-like cells of patients with OD, as estimated by the SOFA score at ICU admission, by APACHE-II 48 and 72 h post-admission, and with survival. The same observation was also made in septic mice for B1b cells and an established clinical severity score. Our results largely follow pre-clinical findings showing that the B1 cell population differs from B2 cells in their phenotype, tissue distribution, and functional characteristics in rodents.^37^^,^^38^ B1 cells represent a major population in the peritoneum and are believed to primarily act as a bridging element between innate and adaptive immunity by spontaneously expressing and secreting low-affinity poly-reactive IgM, thereby contributing to bacterial clearance at early stages of infection.^21^^,^^22^ Our observation of increased peritoneal B1-like lymphocyte percentages during the acute phase of peritonitis appears reasonable from a pathophysiological viewpoint. The fact that we did not detect CD5^+^ B1-like cells in some samples may be explained by the very early time point of sampling after the onset of sepsis. Thus, we may have missed a potential “peak effect” with steep boundaries at earlier or later time points. As B1-like cells remained in the peripheral blood of septic patients despite all other adaptive immune cells being reduced, a migration of B1-like cells between the blood and peritoneum can be suggested. Indeed, B1 cells can migrate from the pleural space to the lung parenchyma in response to lung infection.^39^ As we focused on sepsis patients with peritonitis, we can only speculate that lung tissue could have been involved in the inflammatory response. However, the pleura is, besides the peritoneum, a major site of B1 cell presence in mice.^21^^,^^23^ Further investigations of the pleura were beyond the scope of our study, as we aimed at characterizing early immune cell changes in patients with septic peritonitis undergoing surgery versus surgery-only, and whether the findings in animal models are, in principle, transferrable to human patients.

Few experimental or clinical data are currently available on B1-like lymphocytes and their fate and role in sepsis. Stimulation of B1-like cell populations in early sepsis could result from capillary leakage and impaired microcirculation since it is discussed that hypoxia, mediated by hypoxia-inducible factors, plays a role in B1-like cell regulation.^40^ Recent findings suggested a link between B1-like cells and the pathogenesis of numerous autoimmune diseases such as systemic lupus erythematosus and diabetes mellitus, possibly due to auto-antibody production.^41^^,^^42^ As B1-like cells are a source of anti-inflammatory IL-10,^41^^,^^43^ their function might lie in dampening an inflammatory response. Therefore, we hypothesize that B1-like cells may be a contributor to the early host response to infection in terms of cytokine production and as part of the first defense line by controlling inflammation to combat invading infectious agents effectively. Furthermore, B1-like cells are also involved in the stimulation of T cells.^41^ Thus, an increase in peritoneal B1-like cells could be part of a compensatory mechanism to counterbalance the suppression of adaptive immunity by lymphopenia and other processes. Considering the ability of B1-like cells to express and secrete IgM spontaneously, it appears possible that B1-like lymphocytes have a protective role under sepsis conditions.^39^^,^^44^

In conclusion, our exploratory clinical study shows for the first time increased B1-like cells in the peritoneum of patients suffering from early-stage sepsis due to secondary peritonitis, which are related to OD and prognosis as measured by SOFA and APACHE-II scores at ICU admission. Our findings warrant further investigation of this understudied cell population in severe infections and sepsis.

### Limitations of the study

A major limiting factor is the observational nature of this explorative study, and we can accordingly not provide evidence for mechanisms or causal relationships. Our sample sizes were small, as we only included sepsis patients undergoing open abdominal surgery to eliminate surgery as a confounding factor. Moreover, we only analyzed patients suffering from sepsis due to secondary peritonitis and only a single time point during the acute phase of sepsis, as surgical drains to collect peritoneal fluids were removed after 24–48 h following our clinical guidelines. Moreover, our data are exclusively representative of patients with peritoneal sepsis and with Caucasian background and, therefore, might not be generalizable. Future studies should be designed to circumvent the mentioned bottlenecks and provide longitudinal data for a trajectory during the disease.

## STAR★Methods

### Key resources table

**Table:** 

REAGENT or RESOURCE	SOURCE	IDENTIFIER
**Antibodies**		

anti-B220-APC clone RA3-6B2	eBioscience	cat#17-0452-83; RRID: AB_469394
anti-B220-FITC clone RA3-6B2	BioLegend	cat#103206; RRID: AB_312991
anti-CD11b-FITC clone M1/70.15.11.5	Miltenyi Biotec	cat#130-081-201; RRID: AB_2733615
anti-CD11b-FITC clone M1/70	BioLegend	cat#101206; RRID: AB_312789
anti-CD11b-PE-Cy7 clone M1/70	BioLegend	cat#101216; RRID: AB_312799
anti-CD11b-V450 clone ICRF44	BD Biosciences	cat#560481; RRID: AB_1645556
anti-CD19-APC clone 1D3	BioLegend	cat#152410; RRID: AB_2629839
anti-CD19-FITC clone 1D3	ImmunoTools	cat#22270193
anti-CD19-FITC clone 1D3	BioLegend	cat#101505; RRID: AB_657665
anti-CD19-FITC clone HIB19	BD Biosciences	cat#555412; RRID: AB_395812
anti-CD27-APC clone M-T271	BD Biosciences	cat#558664; RRID: AB_1645457
anti-CD3-APC-Cy7 clone SK7	BD Biosciences	cat#557832; RRID: AB_396890
anti-CD3e-APC clone 145-2C11	BD Biosciences	cat#561826; RRID: AB_10896663
anti-CD3e-PE clone 17A2	eBioscience	cat#12-0031-82; RRID: AB_467053
anti-CD43-PE clone S7	BD Biosciences	cat#553271; RRID: AB_394748
anti-CD45-VioBlue clone REA737	Miltenyi Biotec	cat#130-110-802; RRID: AB_2658222
anti-CD4-FITC clone RM4-5	BioLegend	cat#100509; RRID: AB_312712
anti-CD5-biotin clone 53-7.3	eBioscience	cat#13-0051; RRID: AB_466339
anti-CD5-PE clone UCHT2	BD Biosciences	cat#555353; RRID: AB_395757
anti-CD5-VioBlue clone REA421	Miltenyi Biotec	cat#130-123-287; RRID: AB_2811489
anti-CD8a-PE clone 53-6.7	eBioscience	cat#12-0081; RRID: AB_465530
anti-F4/80-PE clone BM8	Life Technologies	cat#MF48004; RRID: AB_10372666
anti-IgM-PE-Cy7 clone eB121-15F9	eBioscience	cat#25-5890-82; RRID: AB_2573490
anti-Ly-6G (Gr-1)-APC clone RB6-8C5	eBioscience	cat#17-5931-81; RRID: AB_469475
anti-NK-1.1-FITC clone PK136	BioLegend	cat#108706
anti-Siglec F-PE clone REA798	Miltenyi Biotec	cat#130-112-332; RRID: AB_2653439
Streptavidin-PerCP-eFluor710	eBioscience	cat#46-4317-82; RRID: AB_10598530

**Biological samples**		

Human peritoneal fluid samples and blood	University Hospital Jena	N/A
Mouse peritoneal fluid samples, blood, and spleen tissue	University Hospital Jena	N/A
Human feces	University Hospital Jena	Gonnert et al.^45^

**Chemicals, peptides, and recombinant proteins**		

Lipopolysaccharide *E. coli* serotype O111:B4	Sigma-Aldrich	L5293
FcR blocking reagent	Miltenyi Biotec	cat#130-092-575

**Critical commercial assays**		

CD45 MicroBeads	Miltenyi Biotec	cat#130-045-801
LEGENDplex mouse inflammation panel	BioLegend	cat# 740150

**Experimental models: Organisms/strains**		

*Mus musculus* C57BL/6JRj	Janvier Labs, University Hospital Jena	N/A
*Mus musculus* C57BL/6JRj *Aicda*^−/−^	Service Unit for Experimental Biomedicine of Friedrich Schiller University Jena	MGI:2654846, Bello et al.,^29^ Muramatsu et al.^30^

**Software and algorithms**		

FACSDIVA V8.0.1	BD Biosciences	https://www.bdbiosciences.com/
FlowLogic 700.2A	Inivai Technologies	https://www.inivai.com
FlowJo 10	BD Biosciences	https://www.bdbiosciences.com/
Prism 8.0.2	GraphPad Software, Inc.	https://www.graphpad.com

### Resource availability

#### Lead contact

Further information and requests for resources and reagents should be directed to and will be fulfilled by the lead contact, Christian Kosan (christian.kosan@uni-jena.de).

#### Materials availability

This study did not generate new unique reagents.

#### Data and code availability

• All data reported in this paper will be shared by the lead contact upon request.
• This paper does not report original code.
• Any additional information required to reanalyze the data reported in this paper is available from the lead contact upon request.

### Experimental model and study participant details

#### Human participants

The study was approved by the faculty’s ethics review board of the University Hospital Jena (reference 3247-09/11). All subjects or their legal representatives gave written informed consent. All included patients were aged 18 years or older and had not undergone other surgical interventions before enrolment. The gender distribution of the patients was comparable. All patients had a White/Caucasian background. Sepsis and septic shock were defined according to recent criteria.^2^ SOFA and APACHE-II scores were evaluated according to established criteria.^2^^,^^46^ Patients receiving any type of blood cell transfusion during surgery or before harvesting samples were excluded from this study. General exclusion criteria were chemotherapy within the last two months, long-term immune-suppressive treatment, history of organ transplantation, active rheumatoid inflammatory disease, and drug or alcohol abuse (defined as a daily alcohol intake of more than 20 g for females and 40 g for males), pre-existing chronic kidney disease or kidney failure with essential hemodialysis and known liver cirrhosis. Baseline data from standard laboratory, clinical measurements, and clinical follow-up data from the intensive care unit (ICU) treatment were recorded from patients. Whole blood from healthy age- and gender-matched volunteers of comparable gender and age was sampled to compare the acute impact of sepsis and surgery on selected immune cell subtypes in peripheral blood.

#### *In vivo* animal studies

All experiments were performed according to German legislation on the protection of animals and with permission from the official animal welfare committee of Thuringia (TVA registration references 02-010/15, 02-018/15, and UKJ-19-019). All legal specifications regarding European guideline 2010/63/EU were followed.

Male and female wild-type C57BL/6JRj mice of 3-8 months of age were housed in the animal facility at the University Hospital Jena (SPF facility). Mice were maintained under automatically controlled 10-14 h day-night cycles, temperature at 20–24°C, and 50–60% humidity. Animals received a standard diet (Sniff, Soest, Germany) and water *ad libitum*. Mice were kept in groups of three to five mice in pre-sterilized cages enriched with wood shavings and wood pulp. Animals were randomly allocated to experimental and control groups of 3-5 animals in 2–3 independent experiments. For endotoxemia experiments, one cohort of *Aicda*^*-/-*^ mice in a C57BL/6JRj background was analyzed (MGI:2654846).

### Method details

#### Sampling of human blood and peritoneal fluids

Whole blood from sepsis and surgery patients was sampled in EDTA tubes. All samples from patients were taken within 24 h after surgery. Venous blood samples were taken from healthy volunteers between 08:00 and 12:00 AM corresponding to the daytime of sampling in patients. Clinical laboratory parameters were measured in a certified university hospital laboratory. All samples for flow cytometric immune-phenotyping remained on ice until being processed immediately for magnetic bead purification. Peritoneal samples from the sepsis and surgery patients were sequentially filtered through MACS SmartStrainers (100 μm, Miltenyi Biotec, cat#130-098-463 and 70 μm, Miltenyi Biotec, cat#130-098-462). Whole blood and peritoneal liquid samples were then centrifuged for 5 min at 700 x g. The supernatant was discarded, and the cell pellet was resuspended in red blood cell lysis buffer (8.3 g/L ammonium chloride in 0.01 M Tris-HCl pH 7.4) and incubated for 10 minutes at room temperature. PBS was added at ten times the volume and the cells were pelleted for 5 min at 700 x g. This procedure was repeated until the pellet did not contain any red blood cells. Leukocytes were isolated from peritoneal fluid samples by positive magnetic selection using CD45 MicroBeads (Miltenyi Biotec, cat#130-045-801) according to the manufacturer’s protocol.

#### Peritoneal contamination and infection (PCI)

For peritoneal contamination and infection, 3.5 μL/g body weight (b.w.) of diluted human feces or saline was injected (i.p.) into the right lower quadrant of the abdomen of male mice as described before.^45^ All animals received antibiotic treatment with 25 mg/kg b.w. meropenem subcutaneously (s.c.) 8 h after the operation and antibiotic therapy was continued in 12 h intervals for three days. Injections and evaluations of the animals were conducted in a non-blinded fashion. Body weight, survival, and a clinical severity score (CSS), a definitive scoring system from 1, with no signs of illness, to 4, reflecting a severe clinical status,^45^ were monitored twice a day. Animals were euthanized by inhalation of 5% isoflurane 24 h after sepsis induction.

#### Endotoxemia

Endotoxemia was induced in male and female mice by intraperitoneal injection of a mid-lethal dose (12.5 μg/g body weight) of purified lipopolysaccharide (*E. coli* serotype O111:B4, Sigma-Aldrich). Mice were monitored intensively up to 72 h post-induction and received PBS volume substitution. Weight, temperature, and disease severity (scored as for PCI) were recorded two times per day for the duration of the experiment.^47^ Mice were sacrificed by cervical dislocation.

#### Cecal ligation and puncture (CLP)

For cecal ligation and puncture (CLP),^48^^,^^49^ male mice were anesthetized with a combination of isoflurane and butorphanol (1 mg/kg, s.c.) followed by 30% ligation of the cecum and double puncture with a 23 G needle. After the puncture, a small amount of feces was extruded, and the cecum was placed back into the abdominal cavity of the mice. Sham treatment was performed without ligation and puncture. All animals received 0.9% saline (40 ml/kg, i.p.) and imipenem/cilastatin (25 mg/kg, i.p.), starting 6 h after CLP for 3 days every 12 h. Animals were scored two times daily for the first 3 days and then once daily for a total of 10 days. Weight, temperature (Rodent Thermometer BIO-TK8851, Bioseb, France), and disease severity (scored as for PCI) were monitored for the duration of the experiment. On day 10, survivors were sacrificed by cervical dislocation.

#### Mouse tissue collection and sample preparation

Organs were exposed by laparotomy without disrupting the peritoneum, and the abdominal cavity was flushed with PBS supplemented with 10% FBS and 2 mM EDTA. Then, internal organs such as the spleen were collected (for the endotoxemia model). Peripheral blood was taken at the time of the autopsy and supplemented with 500 mM EDTA for flow cytometry (for the PCI and CLP model) or centrifuged for 10 min at 1000 g to separate cells from plasma (for the endotoxemia model). A single-cell suspension was generated from the spleen by gently crushing the organ between frosted microscopy slides. Red blood cells were removed by lysis as described before for human samples. Cell suspensions were filtered through cotton-stuffed glass pipettes and Fc receptors were blocked by incubation with FcR blocking reagent (mouse, Miltenyi Biotec, cat#130-092-575) according to the manufacturer’s protocol.

#### Flow cytometry staining

Single-cell suspensions in PBS were mixed with an antibody cocktail and incubated for 10 minutes at room temperature in the dark according to the manufacturer’s protocol. The combination of antibodies varied according to the species and experimental approach.

Human cells were stained with the following antibodies: anti-CD19-FITC (BD Biosciences, cat#555412, clone HIB19); anti-CD27-APC (BD Biosciences, cat#558664, clone M-T271); anti-CD3-APC-Cy7 (BD Biosciences, cat#557832, clone SK7); anti-CD11b-V450 (BD Biosciences, cat#560481, clone ICRF44); anti-CD5-PE (BD Biosciences, cat#555353, clone UCHT2).

Samples from the PCI model were stained using the following antibodies: anti-CD3e-APC (BD Biosciences, cat#561826, clone 145-2C11) and anti-CD19-FITC (ImmunoTools, Friesoythe, Germany, cat#22270193, clone 1D3), or anti-CD11b-FITC (Miltenyi Biotec, cat#130-081-201, clone M1/70.15.11.5) and anti-F4/80-PE (Life Technologies, cat#MF48004, clone BM8).

Samples from the endotoxemia model were stained using the following antibodies: anti-B220-APC (eBioscience, cat#17-0452-83, clone RA3-6B2), anti-CD3e-PE (eBioscience, cat#12-0031-82, clone 17A2), anti-CD5-biotin (eBioscience, cat#13-0051, clone 53-7.3) in combination with Streptavidin-PerCP-eFluor710 (eBioscience, cat#46-4317-82), anti-CD11b-FITC (Biolegend, cat#101206, M1/70), anti-CD19-FITC (Biolegend, cat#101505, clone 1D3), anti-CD43-PE (BD Biosciences, cat#553271, clone S7), anti-IgM-PE-Cy7 (eBioscience, cat#25-5890-82, clone eB121-15F9).

Samples from the CLP model were stained using the following antibodies: anti-CD4-FITC (BioLegend, cat#100509, clone RM4-5), anti-CD5-VioBlue (Miltenyi Biotec, cat#130-123-287, clone REA421), anti-CD8a-PE (eBioscience, cat#12-0081, clone 53-6.7), anti-CD11b-PE-Cy7 (BioLegend, cat#101216, clone M1/70), anti-CD19-APC or -FITC (BioLegend, cat#152410 or cat#101505, clone 1D3), anti-CD43-PE (BD Biosciences, cat#553271, clone S7), anti-CD45-VioBlue (Miltenyi Biotec, cat#130-110-802, clone REA737), anti-B220-APC (eBioscience, cat#17-0452-83, clone RA3-6B2), anti-B220-FITC (BioLegend, cat#103206, clone RA3-6B2), anti-IgM-PE-Cy7 (eBioscience, cat#25-5890-82, clone eB121-15F9), anti-Ly-6G (Gr-1)-APC (eBioscience, cat#17-5931-81, clone RB6-8C5), anti-NK-1.1-FITC (BioLegend, cat#108706, clone PK136), anti-Siglec F-PE (Miltenyi Biotec, cat#130-112-332, clone REA798).

After the incubation time, excessive unbound antibodies were washed off with PBS. The final cell pellet was resuspended in PBS for flow cytometric analysis and stored at 4°C until analysis.

#### Flow cytometry

All flow cytometric measurements were performed using a LSR Fortessa system or Accuri™ C6 Plus (both BD Biosciences). Data were acquired and processed with FACSDIVA V8.0.1 (BD Biosciences). FSC and SSC signals were acquired to eliminate debris and doublets. The number of recorded events depended highly on the sample quality. Means of recorded events were 238854 for healthy blood sample controls (n=7), 272216 for blood samples (n=21), and 101880 for peritoneal lavage samples (n=15). Data were analyzed with FlowLogic 700.2A (Inivai Technologies) or FlowJo 10 (BD Biosciences). Immune cells were identified with the indicated CD markers. In some cases with insufficient staining quality, human myeloid cells were defined as SSC^high^ and human T cells as CD5^+^CD19^-^.

#### Cytokine measurement

Multiplex measurement of cytokines from plasma samples was conducted with the LEGENDplex mouse inflammation panel to detect 13 cytokines at once (13-plex). The assay was performed according to the manufacturer’s protocol (BioLegend, cat#740150). In brief, diluted plasma samples and serially diluted standards were combined with assay buffer and serially diluted standards were supplemented with Matrix C in a 96-well filter plate. Capture beads were added to each well and incubated for 2 h at RT. After washing off unbound proteins, the detection antibody cocktail was added into each well and kept at RT for 1 h. Streptavidin-PE was subsequently added for 30 min. After an additional washing step, samples were analyzed in buffer using flow cytometry according to the manual.

### Quantification and statistical analysis

Prism 8.0.2 (GraphPad Software, Inc.) was used to perform statistical analyses. Data are given as mean + SEM, if not stated otherwise. Boxes in all boxplots span from 25^th^-75^th^ percentile and whiskers indicate the minimum and maximum values inside the 1.5-times interquartile range above and below first and third quartiles. All correlation plots depict 95% confidence intervals. Outlier analysis of all data was performed using the stringent ROUT algorithm with Q=0.1%. Pearson's correlation coefficient was used for correlation analysis. All used statistical tests are given in the figure legend and *p* values are indicated in the figures.
