# Supplementary material for: Increased peritoneal B1-like cells during acute phase of human septic peritonitis
Source: iScience. 2024 Jun 6;27(7):110133. doi: 10.1016/j.isci.2024.110133 (PMC11231613; doi:10.1016/j.isci.2024.110133)
Supplement: Document S1. Figures S1–S7 [file mmc1.pdf]

iScience, Volume *27*

Supplemental information

Increased peritoneal B1-like cells

during acute phase of human septic peritonitis

Christian von Loeffelholz, René Winkler, Cynthia Weigel, Eva-Maria Piskor, Wolfgang Vivas, Falk Rauchfuß, Utz Settmacher, Ignacio Rubio, Sebastian Weis, Markus H. Gräler, Michael Bauer, and Christian Kosan

Supplemental Figures

Supplemental Figure S1: Gating strategy used to identify immune cell types in human samples. Related to Figure 1 and 2.

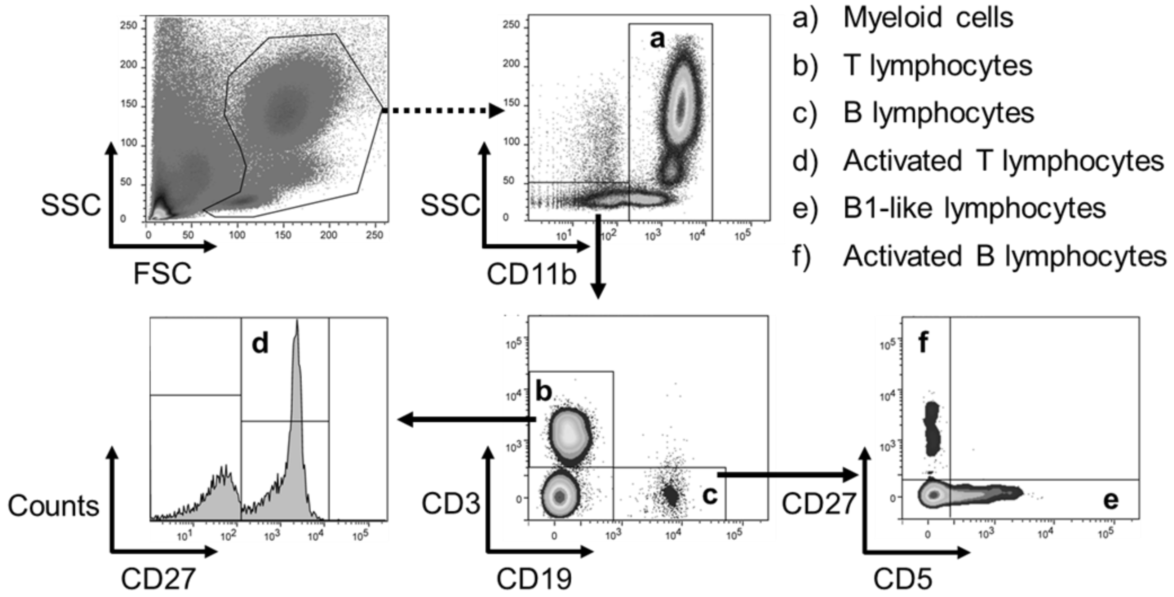

Living cells were pre-gated on FSC and SSC. Debris and doublets were excluded. Markers and immune cell subtypes are indicated.

Supplemental Figure S2: Associations of immune cell percentages in the peripheral blood with fatalities. Related to Figure 3.

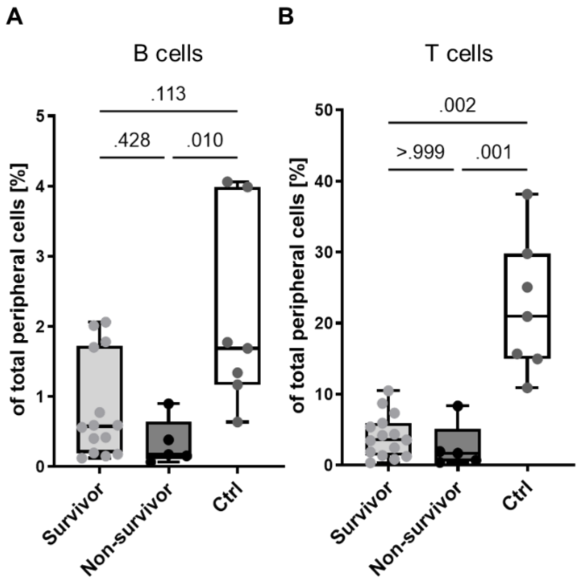

Percentages of peripheral (**A**) B cells and (**B**) T cells were compared between survivors, non-survivors, and controls. Each dot indicates one patient. Kruskal-Wallis test with *post-hoc* Dunn's correction.

Supplemental Figure S3: Extended analysis of mice after peritoneal contamination and infection (PCI) experimental sepsis. Related to Figure 4.

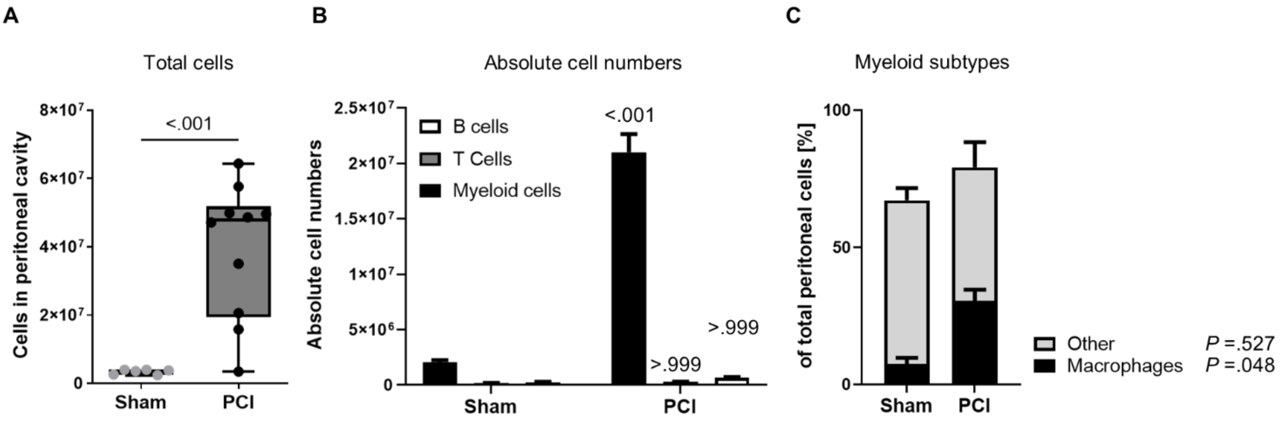

Animals received treatment as described in Figure 4A. (**A**) Total cell numbers in the peritoneal cavity. Each dot indicates one mouse. n=6-10 mice per group. Unpaired, two-tailed t-test. (**B**) Absolute cell numbers of B cells, T cells, and myeloid cells from the peritoneal cavity under the indicated conditions. n=6-10 mice per group. Bars depict mean + SEM. Two-way ANOVA with Bonferroni's correction. (**C**) Myeloid immune cell subtypes in the peritoneal cavity were analyzed. Macrophages were defined as F4/80+. n=6-10 mice per group. Bars depict mean + SEM. Two-way ANOVA with Bonferroni's correction.

Supplemental Figure S4: Extended analysis of mice after endotoxemia (LPS). Related to Figure 4.

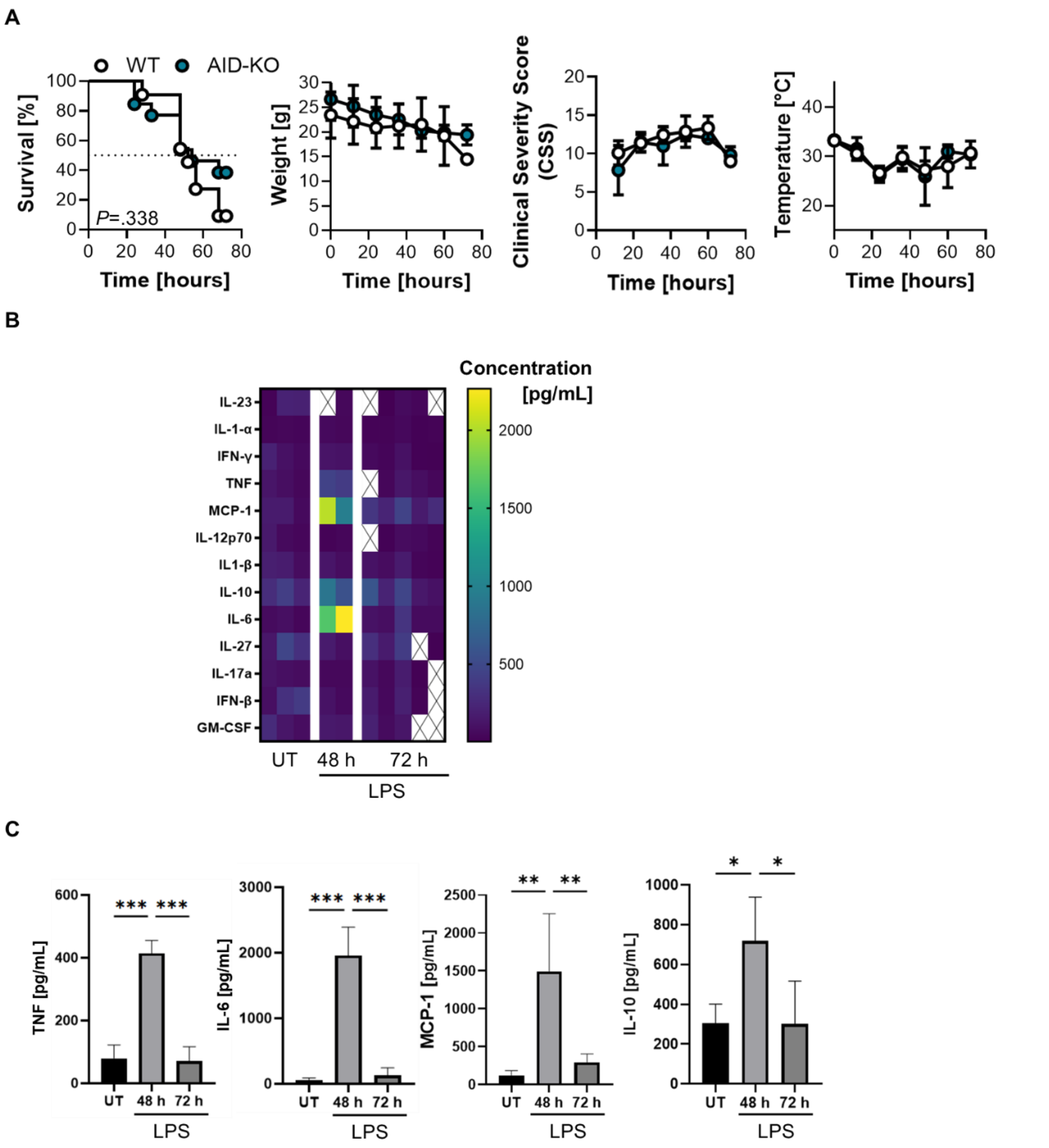

Animals received treatment as described in Figure 4D. (**A**) Parameters of the endotoxemia animal model: survival with Log-rank test, body weight, clinical severity, and body temperature. Dots depict mean ± SEM. A homozygous deletion of *Aicda* characterized AID-KO mice. n=12 mice per group. No differences between wild-type

and AID-KO mice were observed. (**B**) Cytokines in the plasma from peripheral blood were measured using a multiplex flow cytometry approach (LEGENDplex). A blank cell indicates that no value was obtained. (**C**) The four most deregulated cytokines from (**B**) during endotoxemia are shown in bar plots. Bars depict mean + SD as only two samples were available from the 48-h group. One-way ANOVA.

Supplemental Figure S5: Extended analysis of mice after cecal ligation and puncture (CLP) experimental sepsis. Related to Figure 4.

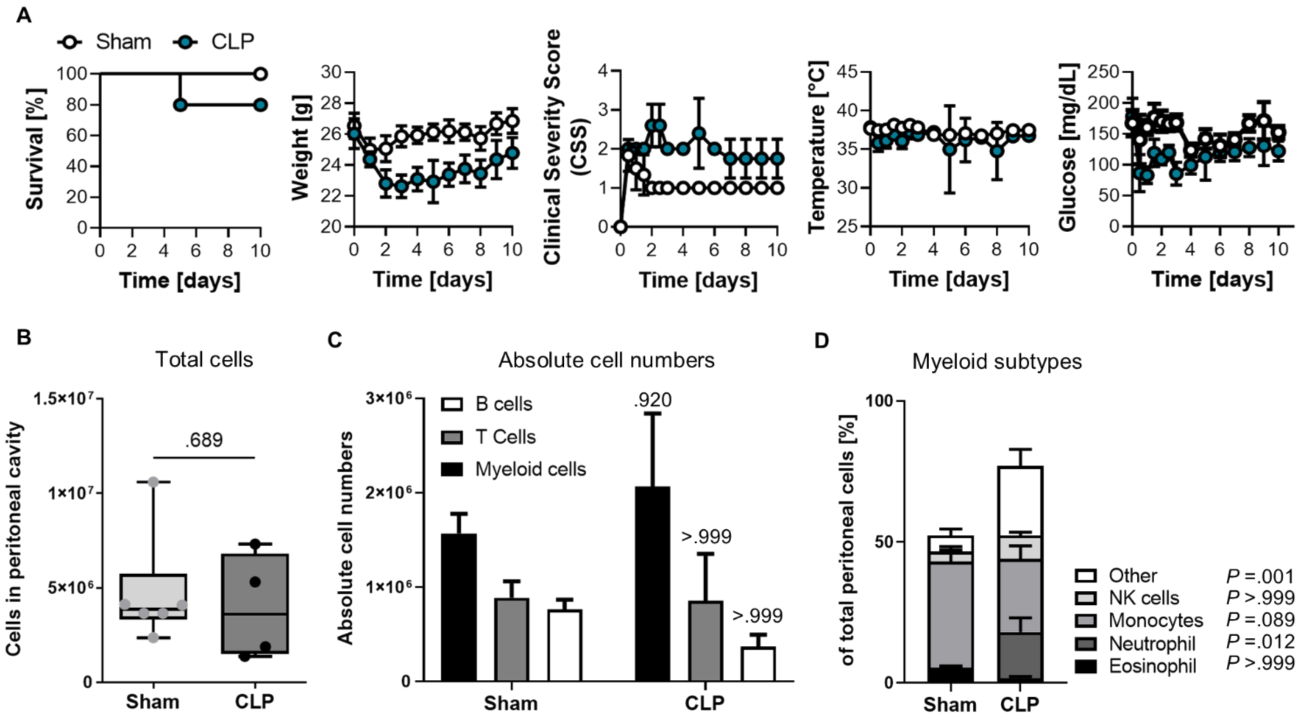

Animals received treatment as described in Figure 4G. (**A**) Parameters of the CLP animal model: survival, body weight, clinical severity, body temperature, and blood glucose levels. Dots depict mean ± SEM. n=5-6 mice per group. (**B**) Total cell numbers in the peritoneal cavity. n=4-6 mice per group. Unpaired, two-tailed t-test. (**C**) Absolute cell numbers of B cells, T cells, and myeloid cells from the peritoneal cavity under the indicated conditions. n=4-6 mice per group. Two-way ANOVA with Bonferroni's correction. (**D**) Myeloid immune cell subtypes in the peritoneal cavity were analyzed by flow cytometry. Cells were defined as the following: NK cells (NK1.1+), Monocytes (CD11b, Gr1-, SiglecF-), Neutrophils (CD11b+, Gr1+), Eosinophils (CD11b+, SiglecF+). n=4-6 mice per group. Bars depict mean + SEM. Two-way ANOVA with Bonferroni's correction.

Supplemental Figure S6: Gating strategy used to identify B1 immune cell subtypes in murine samples. Related to Figure 5.

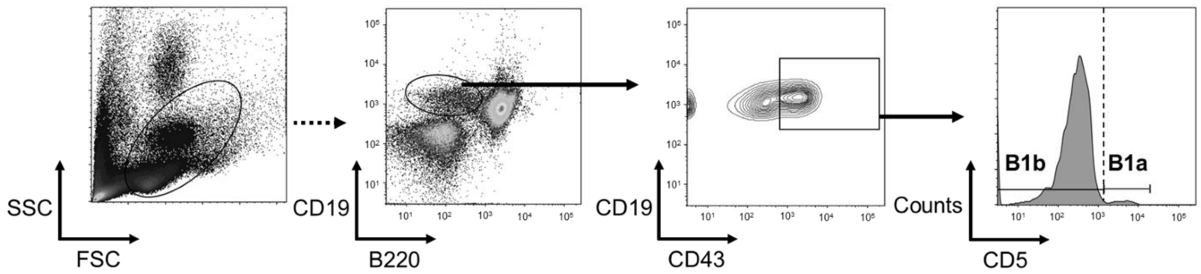

Gating strategy for murine samples from the CLP and endotoxemia experiments. Living cells were pre-gated on FSC and SSC. Debris and doublets were excluded. Markers and immune cell subtypes are indicated.

Supplemental Figure S7: Extended analysis of B1 immune cell subsets in mice after endotoxemia (LPS). Related to Figure 5.

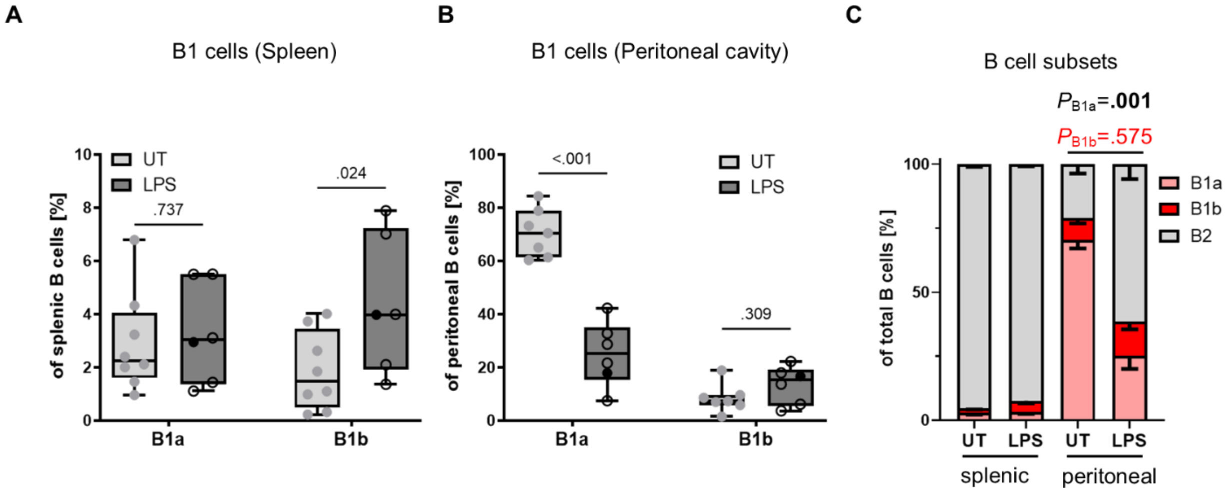

Animals received treatment as described in Figure 4D. B1 cells (CD19+B220lowCD43+) were further distinguished by CD5 into B1a (CD5+) and B1b (CD5-) for spleen (**A**) and (**B**) peritoneal lavage fluid. n≥6 mice per group. Each dot indicates one animal. Open dots indicate mice that were deficient for *Aicda*. Two-Way ANOVA. (**C**) The percentage of B1 and B2 cell populations among total B cells is shown under the indicated conditions. Bars depict mean - SEM. Two-Way ANOVA with *post-hoc* Tukey's correction.
